# Bioabsorbable Suture Anchor Migration to the Acromioclavicular Joint: How Far Can These Implants Go?

**DOI:** 10.1155/2014/834896

**Published:** 2014-07-10

**Authors:** Giovanna Medina, Guilherme Garofo, Caio O. D'Elia, Alexandre C. Bitar, Wagner Castropil, Breno Schor

**Affiliations:** Shoulder and Elbow Service, Vita Institute, Rua Mato Grosso, 306 1 Andar, Higienopolis, 01239-040 São Paulo, SP, Brazil

## Abstract

Few complications regarding the use of bioabsorbable suture anchors in the shoulder have been reported. What motivated this case report was the unusual location of the anchor, found in the acromioclavicular joint which, to our knowledge, has never been reported so far. A 53-year old male with previous rotator cuff (RC) repair using bioabsorbable suture anchors presented with pain and weakness after 2 years of surgery. A suspicion of retear of the RC led to request of a magnetic resonance image, in which the implant was found located in the acromioclavicular joint. The complications reported with the use of metallic implants around the shoulder led to the development of bioabsorbable anchors. Advantages are their absorption over time, minimizing the risk of migration or interference with revision surgery, less artifacts with magnetic resonance imaging, and tendon-to-bone repair strength similar to metallic anchors. Since the use of bioabsorbable suture anchors is increasing, it is important to know the possible complications associated with these devices.

## 1. Introduction

The innovations and improvement in arthroscopic technology enabled new approaches to the treatment of orthopedic conditions, especially the shoulder. One of the most important devices is the suture anchor, which is a small implant that permits fixation of soft tissue to bone and allowed the change from open to arthroscopic procedures.

The first anchors were from metallic materials and opened a range of possibilities in shoulder surgery. However, complications related to their use did not take long to be reported [1, 2]. Among these complications the most reported are breakage, migration, and loosening of the anchor, which can result in devastating premature degenerative arthrosis of the glenohumeral joint.

Bioabsorbable suture anchors were developed as an alternative to metallic anchors [3]. Advantages of the bioabsorbable anchors include their ability of being absorbed over time, with reduction of complications related to migration, and the possibility of performing magnetic resonance imaging (MRI) without the artifact produced by the metal [4]. According to Speer and Warren [3] these implants should have some properties to increase the likelihood of surgical success: (1) they should have initial fixation strength to allow soft tissue to heal onto the bone; (2) material property and time to degradation of the implant must allow satisfactory strength while the healing tissues are regaining mechanical integrity, and (3) they must not degrade too slowly.

However, even bioabsorbable anchors have been causing troubles, and the number of reported complications in the literature is rising with the expanding use of these implants. Most reports in the literature are case reports and case series of complications such as synovitis, glenoid osteolysis, cartilage injury, and anchor breakage [5–8]. What motivated this case report was the unusual location of the anchor, found in the acromioclavicular joint (ACJ) which, to our knowledge, has never been reported so far. Also, the possibility of migration to distant locations should be thought of. Disastrous cases regarding migration of Kirschner wires are famous in the orthopedic field, in which these implants were found in distant locations, sometimes with high risk of injury to vital structures, such as eyes, lungs, great vessels, and spinal cord. This should raise our attention to the possibility that these small materials such as suture anchors can migrate and lodge in distant areas.

## 2. Case Report

We present here the case of a 53-year-old male, right hand dominant, that presented to our clinic with left shoulder pain. He had been operated on on his left shoulder 2 years before, for a rotator cuff (RC) repair with bioabsorbable suture anchors. The patient's physical activity is mainly consisted of cardiovascular rehabilitation that is performed for prior aortic dissection.

Physical examination of the involved shoulder revealed normal passive range of motion (ROM), a discrete pain on palpation of the long head of biceps (LHB) tendon, and weakness for abduction and elevation against resistance. No pain was present on palpation of the ACJ, but the O'Brien test was positive either in pronation or in supination of the upper extremity.

**Figure 1:**
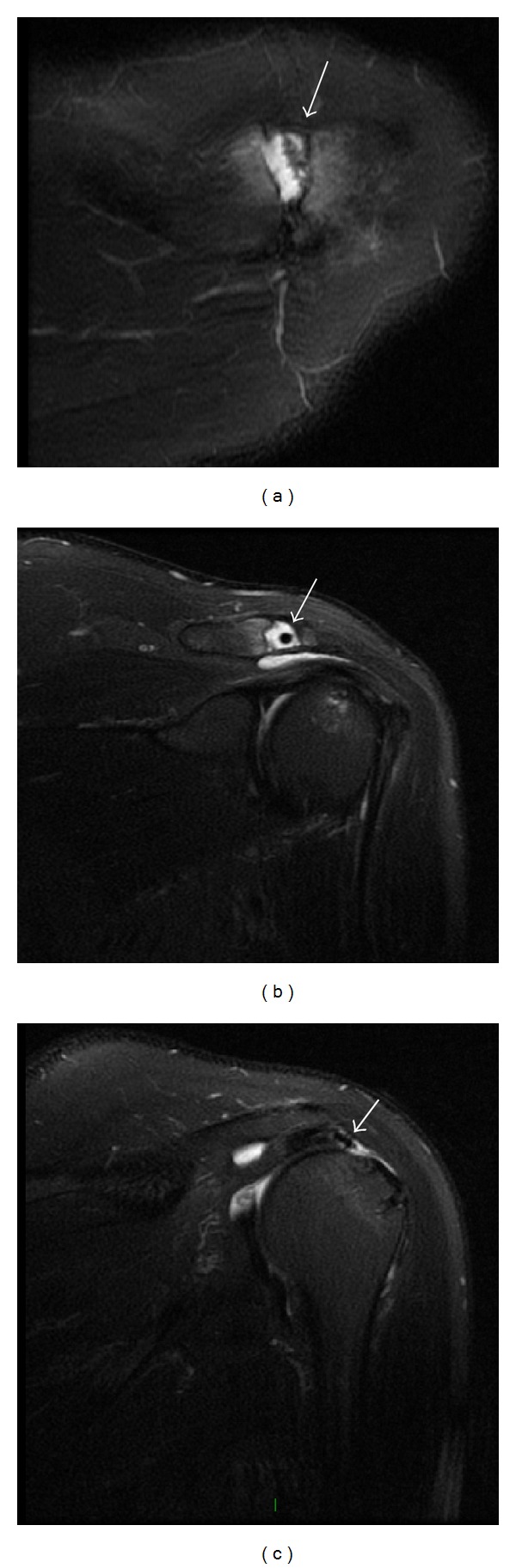
T2-weighted MRI of the left shoulder. (a) Axial view and (b) coronal view showing suture anchor located in the acromioclavicular joint (white arrow); (c) coronal view showing the suture anchor attached to the tendon stump of a retear of the rotator cuff (white arrow).

Because of the suspicion of a retear of the RC, a MRI was requested. This exam revealed bone edema at the distal end of the clavicle and medial aspect of the acromion. A small amount of fluid and a suture anchor were localized intra-articularly in the ACJ (Figures 1(a) and 1(b)).

Other findings were subchondral cysts on the tuberosities and tendinopathy of the supraspinatus tendon with associated complete rupture of this tendon which was retracted for approximately 2.5 cm with the previous suture anchor attached to the tendon stump. Discrete LHB tendinopathy was noted in its intra-articular portion (Figures 1(b) and 1(c)).

**Figure 2:**
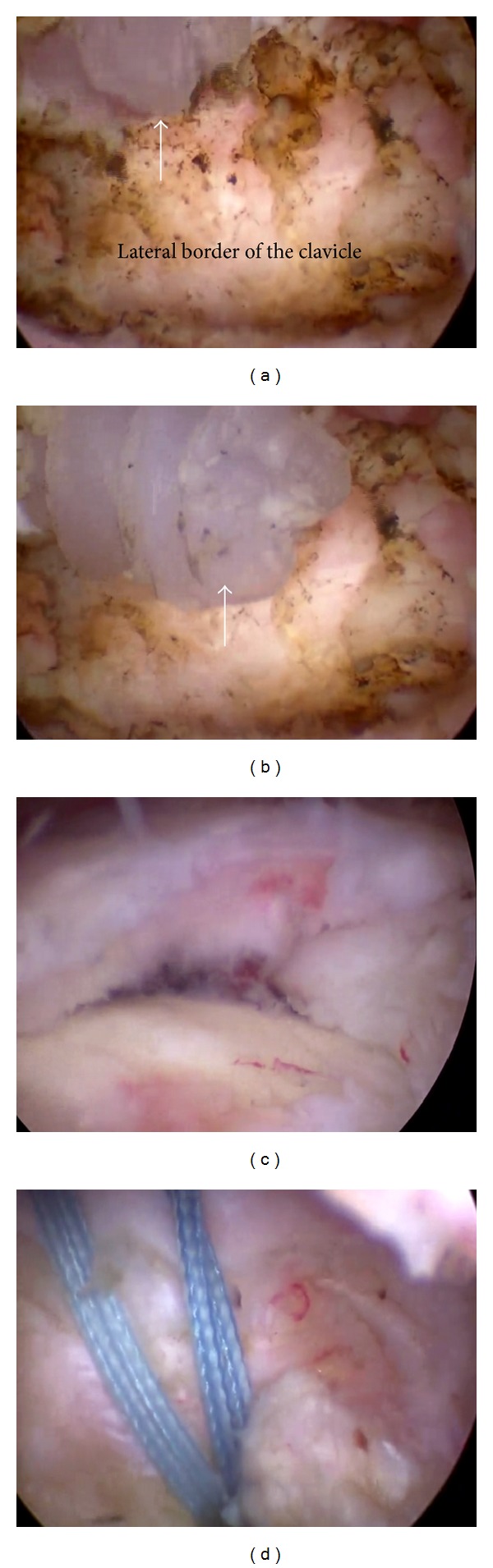
Arthroscopic images confirmed the MRI findings. (a) View through the posterior portal: it is able to see the bioabsorbable anchor in the ACJ (white arrow). We used a spinal needle inserted through the skin superiorly through the ACJ joint, to push the anchor down ((b) white arrow) and retrieve it from the lateral working portal. (c) The retear of the rotator cuff was repaired with double row technique (d) using 2 medial and 1 lateral bioabsorbable anchors.

The patient was then submitted to surgical treatment and all these findings were confirmed with arthroscopy. During the procedure the anchor localized in the ACJ was not overt because of fibrous tissue covering the implant. After removing the soft tissue with a shaver, the bioabsorbable anchor was exposed inside the ACJ joint (Figure 2(a)). The surgeon grasped the small device with a grasper and, to assist in removing it from the joint, a 14G needle was inserted through the ACJ using an outside-in technique. This needle pushed the anchor downward and the surgeon was able to remove it (Figure 2(b)). The second anchor was visible, attached to the tendon stump, and a large retear of the RC was seen (Figure 2(c)). We removed the anchor from the tendon and performed a new double row repair with bioabsorbable anchors and LHB tenodesis with bioabsorbable interference screw (tendon repair seen in Figure 2(d)). No procedure was performed at the ACJ after anchor removal. The patient remained in a sling for six weeks and followed a more restricted rehabilitation program, with passive range of motion only. After twelve weeks he started active exercises and, by the end of the sixth month after surgery, patient was symptom-free and achieved near normal forward elevation of the arm (150°) with good abduction strength against resistance. External rotation with the arm at the side of the body was 50 degrees and internal rotation reached the tenth thoracic vertebra. A new MRI one year after the procedure showed RC healing with implants in place.

## 3. Discussion

Advantages of bioabsorbable anchors are that they have similar fixation strength compared to the metallic anchors; they produce minimal artifacts on MRI which enables postoperative imaging control such as the healing of the soft tissues sutured. Over time, they will dissolve, with resorption time varying depending on the material of the composition. This is beneficial since it avoids future migration or other harmful effects of the presence of a permanent material within the joint or near joint surfaces. However, complications related to bioabsorbable anchors have been reported, such as migration of portions of a bioabsorbable screw into the subacromial space, synovitis, osteolysis, cyst formation, soft tissue inflammation, and implant breakage [9–11].

The concept of the bioabsorbable anchor is to provide the same advantages as the metallic one, allowing soft tissue to heal while it will be degraded slowly. However if the anchor material is absorbed too fast the soft tissue might not have healed yet and the anchor-suture interface will be weekened. This may jeopardize the whole structure and compromise results. Future studies in vivo regarding failure of bioabsorbable anchors are warranted since they differ from in vitro studies as has been reported by Meyer et al. [12].

In this case we imagine that the anchor failed in its interface with the bone of the humeral head, and this is why it was encountered without damage to its structure inside the ACJ. The second anchor might have failed in the same manner, but it remained attached to the tendon stump, avoiding its migration. The diagnosis of loose anchor was made serendipitously, since what elicited the request of a MRI was the symptoms of a failed rotator cuff repair. The patient had pain and weakness; however, the ACJ was asymptomatic. This may raise attention that migration of suture anchors may be underappreciated if it is located in places that cause few symptoms.

Despite these potential complications, bioabsorbable anchors are a very good option for soft tissue surgeries, such as tendon and ligament repair to bone. Because of the many different suture anchors available in the market it is important that the orthopedic surgeon becomes familiar with these devices and before implementing the material in the patient be aware of the manufacture's guidelines and proper insertion techniques. These arthroscopic techniques require that the surgeon has a prior knowledge of the material being used, in order to take advantage of their potential biomechanical properties. Furthermore, one must develop surgical skills through a relatively moderate to long learning curve that will improve the applicability of materials and innovations developed.

What motivated this case report was the unusual location of the anchor, found in the ACJ which, to our knowledge, has never been reported so far. Since the use of bioabsorbable suture anchors is increasing, it is important to know the possible complications associated with these devices.
